# 静注人免疫球蛋白（pH4）治疗B细胞非霍奇金淋巴瘤患者CD20单抗治疗后低丙种球蛋白血症及感染风险的临床研究

**DOI:** 10.3760/cma.j.cn121090-20240918-00353

**Published:** 2025-05

**Authors:** 雪锴 李, 一凡 沈, 德沛 吴, 杨 徐

**Affiliations:** 苏州大学附属第一医院血液内科，国家血液系统疾病临床医学研究中心，江苏省血液研究所，苏州 215006 The First Affiliated Hospital of Soochow University, National Clinical Research Center for Hematologic Diseases, Jiangsu Institute of Hematology, Suzhou 215006, China

**Keywords:** 免疫球蛋白类，静脉内, 低丙种球蛋白血症, B细胞非霍奇金淋巴瘤, CD20单抗, Immunoglobulins, intravenous, hypogammaglobulinemia, B cell non-Hodgkin lymphoma, CD20 monoclonal antibody

## Abstract

**目的:**

观察静注人免疫球蛋白（pH4）（简称IVIg）联合治疗对B细胞非霍奇金淋巴瘤（NHL）患者总免疫球蛋白（Ig）水平的影响，评估IVIg改善B细胞NHL经CD20单抗治疗后低丙种球蛋白血症的临床有效性。

**方法:**

回顾性分析2018年1月至2022年6月期间于苏州大学附属第一医院血液科住院就诊的98例B细胞NHL并使用CD20单抗治疗后低丙种球蛋白血症患者的临床资料。其中，IVIg组70例、常规治疗组28例。为排除输注血浆对总Ig水平的干扰，对未使用血浆IVIg组（53例）和常规治疗组（25例）的总Ig水平进行统计分析。通过观察IVIg对于患者总Ig水平提升效果和持续时间，分析IVIg治疗效果，以及通过比较其他血液生化指标，分析IVIg的控制感染效果。并观察IVIg在临床中应用的安全性。

**结果:**

IVIg组IVIg治疗后1～3 d内总Ig水平高于IVIg治疗前［（20.67±4.17）g/L对（17.16±1.76）g/L，*P*<0.001］。22例患者IVIg治疗后1～7 d、8～14 d、15～30 d的总Ig水平较治疗前差异均具有统计学意义（均*P*<0.001）。常规治疗组住院后1～3 d内总Ig水平均值较入院时差异无统计学意义［（18.12±1.84）g/L对（18.43±1.79）g/L，*P*>0.05］。IVIg组在IVIg治疗后1～3 d内总Ig水平达到20 g/L的患者比例显著高于入院1～3 d内的常规治疗组（57.69％对0，*P*<0.001）。12例入组时中性粒细胞水平低于正常值的IVIg组患者治疗后1～3 d、4～7 d、8～14 d的中性粒细胞水平均较治疗前升高（均*P*<0.05）。IVIg组治疗后新发感染患者比例低于常规治疗组［22.64％（12/53）对36.00％（9/25），*P*>0.05］。IVIg组（70例）中8例患者出现1～2级的不良反应，其中5例出现恶心呕吐、2例出现皮疹、1例肌肉关节痛，未观察到3级以上的不良反应。

**结论:**

IVIg提高B细胞NHL患者CD20单抗治疗后Ig和中性粒细胞水平，对控制新发感染可能有一定作用。IVIg治疗B细胞NHL使用CD20单抗后低丙种球蛋白血症疗效确切且安全性良好。

## Abstract (en)

**Objective:**

To observe the effect of intravenous human immunoglobulin（pH4）（IVIg）on total immunoglobulin（Ig）levels in patients with B-cell non-Hodgkin lymphoma（NHL）and to evaluate its clinical efficacy in ameliorating hypogammaglobulinemia following CD20 monoclonal antibody therapy.

**Methods:**

Clinical data of 98 patients with B-cell NHL who developed hypogammaglobulinemia after CD20 monoclonal antibody therapy and were hospitalized in the Department of Hematology, The First Affiliated Hospital of Soochow University, from January 2018 to June 2022, were retrospectively analyzed. Patients were divided into the IVIg group（*n*＝70）and the conventional treatment group（*n*＝28）. To exclude the interference of plasma transfusion on total Ig levels, statistical analysis was performed on the IVIg group without plasma transfusion（*n*＝53）and the conventional treatment group（*n*＝25）. The therapeutic efficacy of IVIg was analyzed by observing its effect on elevating total Ig levels and the duration of this effect. The infection control efficacy of IVIg was assessed by comparing other blood biochemical parameters. The safety of IVIg in clinical application was also evaluated.

**Results:**

In the IVIg group, the mean total Ig level within 1–3 days after IVIg treatment was（20.67±4.17）g/L, significantly higher than the pre-treatment level of（17.16±1.76）g/L（*P*<0.001）. In 22 patients from the IVIg group, total Ig levels at 1–7 days, 8–14 days, and 15–30 days post-treatment were all significantly different compared to pre-treatment levels（all *P*<0.001）. In the conventional treatment group, the mean total Ig level within 1–3 days after hospitalization showed no significant difference compared to the level at admission［（18.12±1.84）g/L *vs*（18.43±1.79）g/L, *P*>0.05］. The proportion of patients in the IVIg group whose total Ig level reached 20 g/L within 1–3 days post-IVIg treatment was significantly higher than that in the conventional treatment group within 1–3 days after admission（57.69％ *vs* 0, *P*<0.001）. In 12 patients from the IVIg group with baseline neutrophil levels below normal, neutrophil levels at 1–3 days, 4–7 days, and 8–14 days post-treatment were significantly increased compared to pre-treatment levels（all *P*<0.05）. The proportion of patients with new-onset infections post-treatment was lower in the IVIg group（22.64％, 12/53）than in the conventional treatment group（36.00％, 9/25）, although the difference was not statistically significant（*P*>0.05）. Among 70 patients in the IVIg group, 8 patients experienced grade 1–2 adverse reactions, including nausea and vomiting in 5 patients, rash in 2 patients, and muscle/joint pain in 1 patient. No grade 3 or higher adverse reactions were observed.

**Conclusion:**

IVIg increased Ig and neutrophil levels in patients with B-cell NHL after CD20 monoclonal antibody therapy and may play a role in controlling new-onset infections. IVIg is effective and safe for treating hypogammaglobulinemia secondary to CD20 monoclonal antibody therapy in patients with B-cell NHL.

静注人免疫球蛋白（IVIg）是从健康人血浆中分离制得的广谱抗毒素免疫球蛋白（Ig）制剂，具有免疫调理及中和病毒的作用。输注IVIg可迅速提高患者血液中IgG水平，临床上主要用于治疗多种原发性和继发性Ig缺陷症以及部分自身免疫性疾病[1]–[3]。在血液恶性肿瘤中，自身疾病因素以及包括CD20单抗在内的免疫治疗方案都有可能导致低Ig血症的发生。目前关于IVIg治疗继CD20单抗治疗B细胞非霍奇金淋巴瘤（NHL）后低丙种球蛋白血症及控制感染的研究较少。本研究拟通过收集血液科B细胞NHL使用免疫抑制剂后继发低丙种球蛋白血症患者的临床资料，观察IVIg治疗对患者总Ig水平的影响，评估IVIg预防和临床治疗的有效性。

## 病例与方法

一、病例

研究共纳入2018年1月至2022年6月于苏州大学附属第一医院血液科住院就诊的B细胞NHL并使用含CD20单抗方案治疗后继发低丙种球蛋白血症的患者98例。纳入标准：①诊断为B细胞NHL患者；②纳入本研究前1年内已接受CD20单抗［和（或）联合化疗方案］治疗1个疗程及以上；③纳入本研究前存在低丙种球蛋白血症（总Ig水平<20 g/L）。排除标准：①任何形式的非应用免疫抑制剂引起的低丙种球蛋白血症；②严重心脑血管疾病及肝肾功能异常；③怀孕或哺乳；④对人Ig过敏或有其他严重过敏史；⑤IgA抗体的选择性抗IgA缺乏；⑥当次就诊2周内进行了移植、血浆置换等处置。根据是否使用IVIg，将低丙种球蛋白血症患者分为IVIg组和常规治疗组。IVIg组70例，常规治疗组28例。70例患者中1个疗程IVIg使用总量<10 g 11例、10～20 g 43例、>20 g 9例，另有7例患者使用IVIg用量记录缺失。本研究已通过苏州大学附属第一医院医学伦理委员会审批，批件号为（2022）伦研批第285号。本研究为利用以往临床诊疗中的病例的回顾性研究，已申请免除知情同意。

二、观察指标

IVIg组IVIg治疗前3天内总Ig水平和IVIg治疗后1～3 d、4～7 d、8～14 d、15～30 d内总Ig水平以及中性粒细胞水平和感染情况。

三、随访

采用查阅患者住院病历及门诊病历的方式随访，随访起止日期为2018年1月2日至2022年8月15日，中位随访时间：IVIg组为19（13，27）d、常规治疗组为11.5（4，17）d。

四、统计学处理

本研究使用软件R 4.1.2版本及SPSS 26.0软件分析数据。符合正态分布的计量资料以*x*±*s*表示，采用独立样本*t*检验，非正态分布的计量资料以*M*（*Q*1，*Q*3）表示，采用秩和检验，计数资料以例（％）表示，采用*χ*^2^检验或Fisher精确检验。采用Kaplan-Meier法分析感染发生率。*P*<0.05为差异有统计学意义。

## 结果

一、一般资料

本研究纳入IVIg组70例、常规治疗组28例。为排除输注血浆对总Ig水平提高的干扰，对其中53例未使用血浆的IVIg组病例和25例未使用血浆的常规治疗组病例的总Ig水平进行统计分析（表1）。两组患者年龄、性别、体重、入组前CD20单抗联合化疗方案疗程数、末次CD20单抗联合化疗方案治疗结束至本次就诊的时间间隔以及基线总Ig水平差异均无统计学意义（均*P*>0.05）。IVIg组治疗前发生感染的患者比例高于常规治疗组［47.17％（25/53）对4.00％（1/25），*P*<0.001］。平均住院时长IVIg组为（20.40±16.20）d，常规治疗组为（9.88±6.48）d，提示入组的患者中IVIg组整体疾病严重程度高于常规治疗组。

**表1:** 未使用血浆的静注人免疫球蛋白（IVIg）组和常规治疗组患者基线特征

变量	IVIg组（53例）	常规治疗组（25例）	统计量	*P*值
年龄［岁，*M*（*Q*1，*Q*3）］	55（51，66）	58（47，63）	*W*＝682	0.843
男性［例（％）］	37（69.81）	14（56.00）	*χ*^2^=1.4	0.231
体重（kg，*x*±*s*）	64.41±10.39	65.60±9.85	*t*＝−0.5	0.628
治疗前发生感染［例（％）］	25（47.17）	1（4.00）	*χ*^2^＝12.4	<0.001
入组前CD20单抗联合化疗方案疗程数［个，*M*（*Q*1，*Q*3）］	4（2，5）	4（2，6）	*W*＝637	0.786
球蛋白［g/L，*M*（*Q*1，*Q*3）］	18.40（17.00，20.70）	18.70（17.40，19.50）	*W*＝664	0.987

二、IVIg疗效分析

1. 总Ig水平：IVIg组治疗后1～3 d总Ig水平高于治疗前的总Ig水平［（20.67±4.17）g/L对（17.16±1.76）g/L，*P*<0.001］。而常规治疗组治疗后1～3 d内总Ig水平与入院时差异无统计学意义（*P*>0.05）。22例患者IVIg治疗后1～7d、8～14 d、15～30 d的总Ig水平［分别为（23.63±5.70）g/L、（24.08±6.18）g/L、（23.25±5.79）g/L］均高于治疗前的总Ig水平［（18.95±3.83）g/L］，差异均具有统计学意义（均*P*<0.001）。

2. IVIg治疗对患者中性粒细胞的影响：IVIg组IVIg治疗前低于正常中性粒细胞水平的有12例，中性粒细胞水平为（0.86±0.64）×10^9^/L（排除使用血浆和正常值及以上水平的患者），IVIg治疗后1～3 d中性粒细胞水平为（3.65±4.76）×10^9^/L（*P*＝0.028）、4～7 d中性粒细胞水平为（6.04±5.37）×10^9^/L（*P*＝0.004）、8～14 d为（4.37±4.39）×10^9^/L（*P*＝0.046），与治疗前相比均有升高。

IVIg组患者和常规治疗组患者（治疗前中性粒细胞水平<1.8×10^9^/L，且未输注血浆，IVIg组患者12例，常规治疗组8例）治疗前后各阶段中性粒细胞水平的趋势如图1所示，IVIg组患者在接受治疗后1～3 d、4～7 d、8～14 d阶段中性粒细胞水平上升趋势高于常规治疗组，使用IVIg治疗后1～3 d中性粒细胞水平较基线升高中位数为2.25（0.79，3.78）×10^9^/L，常规治疗组升高中位数为1.00（0.05，1.57）×10^9^/L；治疗后4～7 d中性粒细胞水平较基线升高中位数为1.68（0.46，8.70）×10^9^/L，明显高于常规治疗组升高的中位数0.26（0.04，0.63）×10^9^/L（*P*＝0.044）；治疗后8～14 d平均中性粒细胞水平较基线升高中位数为1.10（0.14，2.54）×10^9^/L，常规治疗组升高中位数为0.67（−1.01，0.75）×10^9^/L。

**图1:**
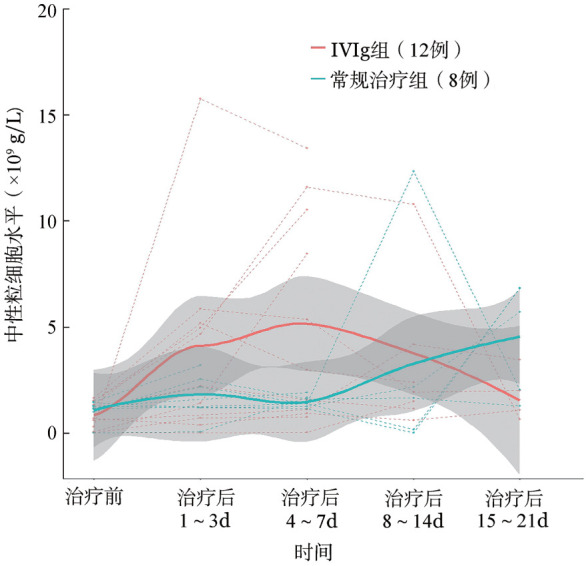
中性粒细胞水平低于正常值的静注人免疫球蛋白（IVIg）组（12例）和常规治疗组（8例）患者治疗前与治疗后各时期中性粒细胞水平趋势图

3. IVIg治疗对目标患者感染等相关症状的短期改善效果：IVIg组患者治疗后有12例发生感染（22.64％，12/53），常规治疗组有9例发生感染（36.00％，9/25），组间差异无统计学意义（*P*＝0.215）。无患者发生二次感染。如图2所示，IVIg组发生新发感染比例在治疗后8 d内均低于常规治疗组。IVIg组在IVIg治疗前有28例未感染，IVIg治疗后3 d内有3例新发感染；常规治疗组入院前有24例未感染，治疗后3 d内有5例新发感染。

**图2:**
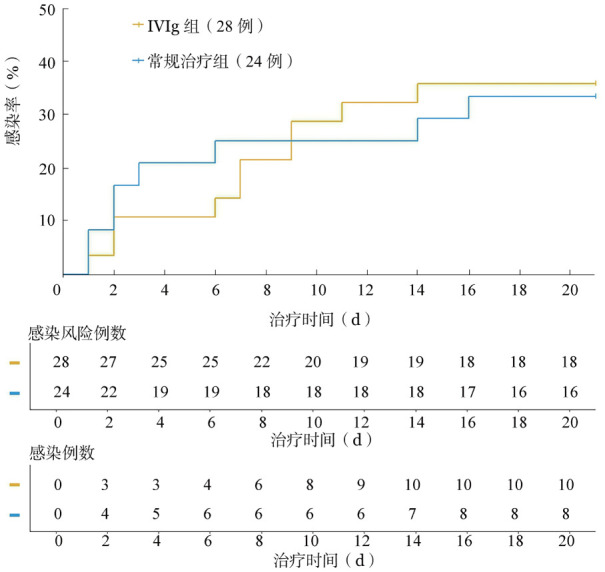
未使用血浆且入院时未发生感染的静注人免疫球蛋白（IVIg）组（28例）和常规治疗组（24例）患者治疗后20 d内感染发生曲线

三、安全性评价

研究记录了入组的使用IVIg治疗的70例患者的相关不良事件，8例患者发生不良事件，均为1～2级，其中5例出现恶心呕吐、2例出现皮疹、1例肌肉关节痛，无低热和心血管系统不良事件发生，因本研究为回顾性，病历中未记载对不良事件原因的判定，研究者也无法再行判定，基于上述情况无法明确这些不良事件与研究药物的关系。本研究记录的不良事件均为1～2级，后续均得到及时处理，提示本研究相关治疗安全性较高。

## 讨论

IVIg是一种从健康人血浆提取的含有广谱抗病毒、细菌或其他病原体的含90％以上IgG抗体的血液制品，具有免疫调理及中和病毒的作用，静脉输注可迅速提高患者血液中IgG水平[1]–[3]。IVIg一直被用于治疗遗传性或原发性免疫缺陷，后被用于获得性Ig缺乏症患者预防感染[4]–[6]。

近年来，各种新疗法包括免疫治疗和分子靶向药物等越来越普遍，其对机体免疫细胞和免疫功能具有独特的、不同程度的影响。在血液系统恶性肿瘤中，自身疾病因素以及包括CD20单抗在内的免疫治疗可能引发低丙种球蛋白血症，而继发性Ig水平降低是血液恶性肿瘤患者易感染的主要原因[7]–[11]。研究对833例低Ig血症患者进行类型分析后发现低球蛋白血症以低丙种球蛋白即IgG最为常见[12]。低水平IgG与感染的频率及严重程度直接相关[13]。英国针对反复出现细菌感染的低丙种球蛋白血症患者的指南[14]提出2个主要策略：抗生素预防和Ig替代治疗。

血液恶性肿瘤如慢性淋巴细胞白血病（CLL）等因继发性低丙种球蛋白血症而发生感染的发生率较高[15]–[20]，Ig替代治疗显著改变了丙种球蛋白缺乏症的临床进程，降低感染发生率[18],[21]。识别继发性低丙种球蛋白血症严重或复发感染的高风险患者，并对其进行适当和预防性治疗，在预防严重感染方面发挥着关键作用。对于获得性低丙种球蛋白血症，国外相关指南推荐[22]：评估血清IgG，如果血清IgG<5 g/L，每个月IVIg 0.3～0.5 g/kg或每周给予适当的皮下免疫球蛋白（SCIG）产品进行替代治疗，调整Ig替代治疗的剂量和间隔以维持血清IgG约5 g/L的最低水平。国内关于血液肿瘤免疫及靶向药物治疗后继发性低丙种球蛋白血症的共识推荐[10]对于IgG≤4 g/L（低丙种球蛋白血症）且合并严重或反复感染的患者，推荐IgG替代治疗（5 g×3 d）。NHL患者治疗期间需要定期输注IVIg以增强免疫力、预防感染，IgG≤4 g/L或复发性感染、严重感染的NHL患者，每3～4周输注1次IVIg，每次0.4 g/kg[23]。

本研究IVIg组患者IVIg治疗后1～3 d内总Ig水平高于IVIg治疗前。IVIg组在IVIg治疗后1～3 d内总Ig水平达到20 g/L的患者比例高于常规治疗组（57.69％对0），验证了IVIg治疗B细胞NHL经CD20单抗治疗后继发低丙种球蛋白血症的有效性，提示IVIg治疗后1～3 d内即可迅速且显著提高患者总Ig水平。本研究观察到IVIg组IVIg治疗后4～7 d、8～14 d、15～30 d，患者的总Ig水平相较于IVIg治疗前仍有提高，说明IVIg治疗对于提高总Ig水平的效果具有一定的持续性，可达到3周左右，与药品说明书介绍的半衰期相一致。

IVIg组和常规治疗组初始中性粒细胞水平差异无统计学意义，IVIg组患者在IVIg治疗后中性粒细胞水平呈缓慢上升趋势且在7 d内均高于常规治疗组。中性粒细胞水平低于正常值的IVIg组患者在IVIg治疗后1～3 d的中性粒细胞水平高于治疗前的中粒细胞水平，且在IVIg治疗后4～7 d与治疗前相比仍有提高。CD20单抗等药物可能导致中性粒细胞水平降低，而中性粒细胞减少症是低丙种球蛋白血症和其他免疫失调状态的临床特征。因此，推测患者输注IVIg后，免疫功能得到一定程度的恢复，继而中性粒细胞也恢复到正常水平[24]–[26]。

IVIg治疗组治疗后新发感染的患者比例低于常规治疗组［22.64％（12/53）对36.00％（9/25），*P*>0.05］。尽管IVIg组患者基础疾病严重程度较重，经IVIg治疗后患者新发感染的风险相较常规治疗组降低，但仍需进一步研究证实差异性。对于两组患者各时间点的感染发生率进行统计，IVIg组和常规治疗组均在治疗后8～14 d和15～21 d两个时间段内新发感染的比例最高，结合两组患者的Ig变化趋势，推测患者新发感染风险在治疗后8～21 d升高与总Ig水平下降相关。建议定期使用IVIg维持总Ig水平，降低患者新发感染风险。

综上，IVIg治疗B细胞NHL使用CD20单抗治疗后继发低丙种球蛋白血症疗效确切，可在1～3 d内即可迅速且显著提高患者总Ig水平，并具有预防低丙种球蛋白血症患者新发感染的趋势。本研究也收集了临床上患者应用IVIg的具体用法用量，发现IVIg用法用量不一，且有些患者存在用量记录缺失的情况，鉴于本研究入组例数较少，随访时间较短，受回顾性临床研究的限制，确切结论尚需大样本的前瞻性研究进一步证实IVIg合理的用法用量以及在IgG水平较低的自身免疫水平低下患者中抗感染的作用。

## References

[1] 陕西省川崎病诊疗中心, 陕西省儿童内科疾病临床医学研究中心, 陕西省人民医院儿童病院 (2021). 静脉输注免疫球蛋白在儿童川崎病中应用的专家共识[J]. 中国当代儿科杂志.

[2] 周 欢, 陈 雨霏, 陈 筱青 (2021). 静脉注射丙种球蛋白在新生儿疾病中的应用[J]. 中华全科医学.

[3] 程 梁华, 彭 莉, 伍 晓晓 (2021). 注射用人免疫球蛋白临床应用研究现状[J]. 中国临床药理学杂志.

[4] 广东省医师协会儿科医师分会, 《中国当代儿科杂志》编辑部 (2021). 静脉注射用免疫球蛋白在儿童血液/肿瘤性疾病中应用的儿科专家共识[J]. 中国当代儿科杂志.

[5] Grigoriadou S, Clubbe R, Garcez T (2022). British Society for Immunology and United Kingdom Primary Immunodeficiency Network (UKPIN) consensus guideline for the management of immunoglobulin replacement therapy[J]. Clin Exp Immunol.

[6] Compagno N, Malipiero G, Cinetto F (2014). Immunoglobulin replacement therapy in secondary hypogammaglobulinemia[J]. Front Immunol.

[7] Fischer T, Ni A, Bantilan KS (2022). The impact of anti-CD20-based therapy on hypogammaglobulinemia in patients with follicular lymphoma[J]. Leuk Lymphoma.

[8] Sim B, Ng JY, Teh BW (2023). Immunoglobulin replacement in hematological malignancies: a focus on evidence, alternatives, dosing strategy, and cessation rule[J]. Leuk Lymphoma.

[9] Labrosse R, Barmettler S, Derfalvi B (2021). Rituximab-induced hypogammaglobulinemia and infection risk in pediatric patients[J]. J Allergy Clin Immunol.

[10] 中华医学会血液学分会感染学组, 中华医学会血液学分会淋巴细胞疾病学组, 中国临床肿瘤学会(CSCO)抗淋巴瘤联盟 (2021). 血液肿瘤免疫及靶向药物治疗相关性感染预防及诊治中国专家共识(2021年版)[J]. 中华血液学杂志.

[11] Ar MC, El Fakih R, Gabbassova S (2023). Management of humoral secondary immunodeficiency in hematological malignancies and following hematopoietic stem cell transplantation: Regional perspectives[J]. Leuk Res.

[12] 马 俊杰, 隋 晶蕊, 陈 明清 (2014). 833例低免疫球蛋白血症类型及疾病分析[J]. 实用临床医药杂志.

[13] Athni TS, Barmettler S (2023). Hypogammaglobulinemia, late-onset neutropenia, and infections following rituximab[J]. Ann Allergy Asthma Immunol.

[14] Oscier D, Fegan C, Hillmen P (2004). guideline Guidelines on the diagnosis and management of chronic lymphocytic leukaemia[J]. Br J Haematol.

[15] Mikulska M, Oltolini C, Zappulo E (2024). Prevention and management of infectious complications in patients with chronic lymphocytic leukemia (CLL) treated with BTK and BCL-2 inhibitors, focus on current guidelines[J]. Blood Rev.

[16] Noto A, Cassin R, Mattiello V (2023). Should treatment of hypogammaglobulinemia with immunoglobulin replacement therapy (IgRT) become standard of care in patients with chronic lymphocytic leukemia?[J]. Front Immunol.

[17] Morrison VA (2010). Infectious complications of chronic lymphocytic leukaemia: pathogenesis, spectrum of infection, preventive approaches[J]. Best Pract Res Clin Haematol.

[18] Soumerai JD, Yousif Z, Gift T (2024). IgG testing, immunoglobulin replacement therapy, and infection outcomes in patients with CLL or NHL: real-world evidence[J]. Blood Adv.

[19] Tsiodras S, Samonis G, Keating MJ (2000). Infection and immunity in chronic lymphocytic leukemia[J]. Mayo Clin Proc.

[20] Wadhwa PD, Morrison VA (2006). Infectious complications of chronic lymphocytic leukemia[J]. Semin Oncol.

[21] Raanani P, Gafter-Gvili A, Paul M (2008). Immunoglobulin prophylaxis in hematological malignancies and hematopoietic stem cell transplantation[J]. Cochrane Database Syst Rev.

[22] Wierda WG, Brown J, Abramson J S (2022). NCCN guidelines® insights: Chronic Lymphocytic leukemia/small Lymphocytic lymphoma, version 3.2022: Featured updates to the NCCN Guidelines[J]. Journal of the National Comprehensive Cancer Network.

[23] 中国抗癌协会肿瘤与微生态专业委员会血液学组, 广东省预防医学会血液肿瘤防治专业委员会, 中国感染免疫与微生态研究转化协作组 (2024). 静脉注射用免疫球蛋白在血液系统疾病中应用的专家共识[J]. 中南药学.

[24] Atarod L, Aghamohammadi A, Moin M (2007). Successful management of neutropenia in a patient with CD40 ligand deficiency by immunoglobulin replacement therapy[J]. Iran J Allergy Asthma Immunol.

[25] Walkovich K, Connelly JA (2021). Understanding neutropenia secondary to intrinsic or iatrogenic immune dysregulation[J]. Hematology Am Soc Hematol Educ Program.

[26] Banatvala N, Davies J, Kanariou M (1994). Hypogammaglobulinaemia associated with normal or increased IgM (the hyper IgM syndrome): a case series review[J]. Arch Dis Child.

